# Nicotine Risk Education and Its Impact on Knowledge, Perceptions, and Behavioral Intentions: A Scoping Review of U.S. Studies

**DOI:** 10.3390/ijerph23050636

**Published:** 2026-05-11

**Authors:** Rabia Imran, Aashka Patel, Morgan Snell

**Affiliations:** 1 Department of Health Policy, Virginia Commonwealth University, Richmond, VA 23284, USA; imranr@vcu.edu (R.I.); patelas13@vcu.edu (A.P.); 2 Department of Biology, Virginia Commonwealth University, Richmond, VA 23284, USA

**Keywords:** nicotine education, relative risk, smoking, harm reduction

## Abstract

**Highlights:**

**Public health relevance—How does this work relate to a public health issue?**

- Reducing the health harms associated with smoking may include strategies such as encouraging smokers who are not yet ready or willing to quit to switch to a lower harm source of nicotine.
- Harm reduction policies will only be successful if adults who smoke understand that nicotine products exist on a continuum of risk, and evidence suggests misperceptions are pervasive.

**Public health significance—Why is this work of significance to public health?**

- Investigating whether education about nicotine’s absolute and relative potential for health harm has the potential to influence perceptions, behavioral intentions to switch products, and actual use behaviors is critical to inform policy.
- An emerging body of work provides robust evidence that nicotine educational messages have the potential to increase knowledge and reduce misperceptions across populations, however, findings are less clear about the role of exposure to nicotine education and nicotine use behaviors.

**Public health implications—What are the key implications or messages for practitioners, policy makers and/or researchers in public health?**

- A range of measures, methods, and endpoints are being employed by investigators exploring nicotine education as an intervention to reduce smoking behavior in the U.S.
- While promising to correct misperceptions about nicotine’s absolute and relative risks across products, more robust evidence on the impact of education on product use is crucial, as is evaluating any unintended consequences of relative risk education.

**Abstract:**

Smoking combustible cigarettes causes an enormous health and financial burden in the U.S. Across tobacco and nicotine products, cigarettes are ranked as the most toxic. Any harm reduction efforts rely on smokers understanding nicotine’s absolute and relative health risk potential, but many studies reveal widespread misperceptions. To inform policies focused on reducing the public health burden of smoking, it is essential to understand whether conveying accurate absolute and relative risk information about nicotine and its delivery methods may shift risk perceptions and impact use behaviors. To fill this gap, we conducted a systematic search, using PubMed, PsycINFO, and Google Scholar, for original U.S. studies investigating effects of exposure to educational messages about (1) nicotine’s addictive and health risk properties or (2) relative risk information on participants’ nicotine knowledge, perceptions, behavioral attentions, and/or use behaviors. Across studies of predominantly fair methodological quality, message exposure was consistently associated with improvements in knowledge and risk perceptions; however, findings were mixed regarding behavioral intentions, and the evidence base is limited by short-term experimental designs without longitudinal follow-up. Our results highlight the potential for educational interventions to increase nicotine knowledge more broadly and reveal important considerations for using education to try to shift behavior among individuals who smoke.

## 1. Introduction

Cigarette smoking remains the leading cause of preventable morbidity and mortality globally, including in the United States (U.S.). Smoking cigarettes accounts for more than 480,000 deaths annually in the U.S. and imposes an estimated $600 billion in combined health care expenditures and lost productivity each year [1]. The immense disease burden is primarily driven by the combustion of tobacco, which produces thousands of toxicants and carcinogens that contribute to cardiovascular disease, pulmonary illness, and multiple cancers [2]. Nicotine itself, while highly addictive, is not the primary source of tobacco-related disease; rather, the method of nicotine delivery plays a crucial role [3]. In this context, “nicotine education” refers to public health or clinical communication efforts designed to improve understanding of nicotine’s pharmacological effects, as well as its role in addiction and disease risk [3,4]. “Relative risk messaging refers to communication strategies that explicitly compare the health risks of different nicotine delivery products. The “continuum of risk” describes the evidence-based spectrum of harm across nicotine products, with combustible cigarettes at the highest risk and noncombustible products at lower risk [3,4].

Previous research examining the nicotine continuum of risk among various tobacco and/or nicotine products placed combustible cigarettes as the most harmful and toxic form of nicotine delivery, whereas noncombustible products such as nicotine replacement therapies (NRTs), electronic nicotine delivery systems (ENDS), and other alternative non-combustible nicotine products were considered to pose substantially lower health risks [3,4]. Despite these differences, misconceptions persist regarding nicotine’s role in disease. Although approximately two thirds of all smokers report a desire to quit, and the prevalence of cigarette smoking among U.S. adults has steadily declined over the past several decades [5,6,7], a significant proportion of smokers remain unable or unwilling to quit entirely due to the addictive properties that cause high nicotine dependence. For these individuals, switching completely to a less harmful source of nicotine could meaningfully reduce exposure to toxicants from cigarette smoke; however, such harm reduction relies on smokers’ accurate understanding of the continuum of risk across nicotine-containing products [8].

Research consistently demonstrates that many U.S. adults, including both smokers and nonsmokers, hold significant misperceptions about nicotine’s role in disease and the relative risks of various nicotine delivery products [9]. Many incorrectly believe that nicotine itself causes most of the cancers and cardiovascular harms associated with smoking, and few accurately recognize that products exist along a continuum of risk, with combustible cigarettes being the most harmful and noncombustible products generally posing substantially lower risks [3]. These misperceptions appear to be more prevalent among specific demographic groups and members of racial and ethnic minority communities such as the Black/African American community and the Hispanic/Latino population [10,11]. Importantly, holding these inaccurate beliefs about nicotine may reduce the likelihood that smokers will attempt to quit or adopt less harmful alternatives such as NRTs or ENDS to aid cessation or replace cigarettes [8]. Improving public understanding of nicotine and the continuum of risk across products could therefore play a critical role in supporting harm-reduction strategies and promoting complete transition away from combustible tobacco use [3].

In the United States, healthcare-based strategies to prevent tobacco use and promote smoking cessation stem from evidence-based clinical guidelines, pharmacological therapies, behavioral counseling, and national public health campaigns, including interventions through the FDA. Clinical practice guidelines often recommend systematic identification of tobacco use, brief clinician counseling, and cessation medications as standard care [12]. While these approaches have improved quit and cessation rates, persistent misperceptions about nicotine risk may limit their effectiveness, particularly when patients are reluctant to use nicotine-containing cessation aids due to safety concerns [13].

Many adult smokers are not completely switching to lower-risk nicotine products; instead, a substantial number of smokers continue to “dual-use,” meaning that they combine the use of cigarettes and e-cigarettes, or do not use alternative products at all. According to recent CDC data, about 29.4% of U.S. adults who vape are also cigarette smokers, a pattern particularly apparent in older age groups; for example, 42.7% of adult smokers older than 45 are dual-users [14]. Among combustible-cigarette smokers who reported a quit-attempt in the past year, only 13.1% were current e-cigarette users in 2020 [15]. Dual-use also varies by age group, with younger adults being more likely to be dual-users than older adults [16]. In terms of quit-methods, 2022 NHIS data showed that 53.9% of U.S. adults who recently stopped or tried to stop smoking used nicotine-containing products, with around 40.8% dual-using e-cigarettes and 26.0% using only e-cigarettes [17]. These patterns suggest that many smokers do not fully understand and/or use the benefits of less harmful nicotine sources, which highlights the need for targeted education to reach these subgroups of smokers.

Importantly, the relationship between educational messaging and behavioral intentions differs across populations, particularly between adults and youth. Among adults, exposure to accurate nicotine education and relative risk messaging has been associated with increased intentions to quit smoking or switch to less harmful products [18]. In contrast, among youth, messaging must be carefully designed, as information about reduced-risk products may influence perceptions and potentially increase curiosity or susceptibility to experimentation if not appropriately framed [19]. The existing evidence base is therefore disproportionately focused on adults, with comparatively limited research examining how such messaging influences adolescent beliefs and behavioral intentions [20].

Public health education campaigns have played a historical role in reducing smoking rates and shaping public comprehension of tobacco-related harms [19]. Large-scale efforts, such as the FDA’s The Real Cost campaign and the Truth Initiative’s, have communicated the risks associated with combustible cigarettes and vaping to the public [21]. However, the messaging directed at smokers has largely focused on the addictive properties of nicotine, while warning labels and public communications rarely address the limited role of nicotine in many smoking-related diseases, as well as the substantial differences in risk across the variety of nicotine delivery products [22]. In order to better inform the development of education efforts, investigators have begun the process of designing and testing targeted interventions aimed at correcting misperceptions about nicotine’s absolute health risks and the relative risks of nicotine products [23]. The critical evaluation of the content, outcomes, study populations, and delivery strategies of these interventions provides critical insights into which strategies can effectively improve smokers’ understanding of the continuum of risk of nicotine products [20]. Such insights highlight the gaps in evidence and messaging that must be addressed in order to successfully implement a large-scale educational campaign aimed at reducing smokers’ misconceptions about various nicotine-containing products [24].

## 2. Materials and Methods

Given the limited body of research on this topic, a scoping review was conducted in accordance with the PRISMA-ScR guidelines [25]. The protocol for this scoping review was registered on the Open Science Framework (OSC) on 2 April 2026; it can be accessed at [https://doi.org/10.17605/OSF.IO/8GKWR], accessed on 8 January 2026. The Preferred Reported Items for Systematic Reviews and Meta-Analysis extension for Scoping Reviews (PRISMA-ScR) Checklist [25] is available in Supplementary Materials Table S1.

### 2.1. Search Strategy

The search strategy was developed by the Principal Investigator (MS) in collaboration with the co-authors (RI and AP). A comprehensive systematic search was conducted utilizing PubMed, PsycINFO, and Google Scholar to identify and synthesize existing literature on the effects of nicotine educational interventions on accurate risk perceptions and the shaping of future behavioral intentions and use behaviors among youth and adults in the U.S.

A combination of Medical Subject Heading (MeSH) terms and topic-specific keywords was used to maximize the retrieval of relevant study articles. The search terms were modified to accommodate the specific requirements of each individual database. An example search string used for PubMed is as follows: (“Electronic Nicotine Delivery Systems”[MeSH] OR “e-cigarette”[tiab] OR ENDS[tiab] OR vape[tiab]) AND (“Harm Reduction”[MeSH] OR “relative risk”[tiab] OR “comparative risk”[tiab] OR “continuum of risk”[tiab]) AND (“Intention”[MeSH] OR “behavioral intention”[tiab] OR “quit intention”[tiab] OR “Smoking Cessation”[MeSH]) AND (“Health Education”[MeSH] OR education[tiab] OR “health message”[tiab] OR message[tiab] OR “public health campaign”[tiab]). The initial search was conducted in September 2024 and updated in March 2026. When performing the search, filters were applied to exclude any articles published before 2014. The full search strategy for all databases can be found in Supplementary Materials Table S2.

### 2.2. Eligibility Criteria

The eligibility criteria for selected articles were as follows: original U.S. studies carried out in the last 12 years (2014–2026) targeting either youth and/or adults, with a research design in which at least a portion of participants were introduced to some form of educational intervention or messaging regarding nicotine or highlights the comparative risks among various tobacco and/or nicotine products. In addition, studies were required to examine the impact of the educational intervention that participants were exposed to, such as changes in knowledge, beliefs, perceptions, behavioral intentions, and/or actions. Lastly, review articles and studies in which data collection was incomplete were excluded to ensure the quality of results.

### 2.3. Study Selection

Upon the completion of literature compilation, all articles captured by the parameters based on search terms were screened for adherence with the inclusion criteria by the two authors (R.I. and A.P.). All potentially eligible records, and any unclear designations, flagged for review by all authors (R.I., A.P., and M.S.). Full-text eligibility assessment was conducted by R.I. and A.P., with any discrepancies resolved through discussion among the authorship team (R.I., A.P., and M.S.), ensuring compliance with the inclusion criteria.

The majority of records excluded at title and abstract screening did not meet one or more of the following criteria: U.S.-based study population, experimental or quasi-experimental design involving direct exposure to educational messaging, and outcomes measuring knowledge, perceptions, or behavioral intentions related to nicotine or tobacco products. Studies addressing tobacco cessation pharmacotherapy, international populations, or epidemiological patterns without an educational messaging component were the most common reasons for exclusion.

### 2.4. Data Charting

Two reviewers (R.I. and A.P.) extracted data from eligible studies using a standardized data extraction sheet. The following information was charted: author(s) and year of publication, population, study design, education focus, study outcomes, and major findings. Specific data items facilitated the mapping of the extracted data, such as shift in participants’ knowledge and the perceived relative harm of non-combustible tobacco products compared to combustible tobacco products. Additional data items captured behavioral intentions, such as the willingness to switch to less harmful nicotine and/or tobacco products. Discrepancies during data charting process were resolved through consultation with the senior author (M.S.).

### 2.5. Quality Assessment

Given that this review draws conclusions relevant to the design of future educational interventions, a formal quality appraisal was conducted for all 17 included studies, supplementing the standard scoping review approach. Two authors (RI and AP) independently assessed each study using the NIH Study Quality Assessment Tool [26] for Controlled Intervention Studies for randomized and non-randomized experimental designs, and the NIH Quality Assessment Tool [26] for Before-After (Pre-Post) Studies for Leavens et al. (2021), the sole pre-post design in the review [27]. Each criterion was rated as Yes (Y), No (N), Not Applicable (N/A), or Unclear (?). Overall quality ratings of Good, Fair, or Poor were assigned following NIH guidance [26].

The overall study quality ratings (Good, Fair, Poor) were assigned based on the number of “No” responses to the pre-determined high-priority questions (Q1, Q2, Q6, Q9, Q12) from the NIH study quality assessment framework. Studies were rated as “Good” when all high-priority questions were marked as “Yes”. A rating of “Fair” was assigned when at most one high-priority question was marked as “No” and unclear. Studies were rated as Poor when two or more high-priority items were marked “No”, indicating a higher risk of bias. This approach was utilized to ensure consistency with the risk of bias assessment and emphasize the impact of key methodological limitations on overall study validity. Disagreements were resolved through discussion with the senior author (M.S.). Results of the quality appraisal are presented in Supplementary Materials Table S3.

## 3. Results

**Figure 1:**
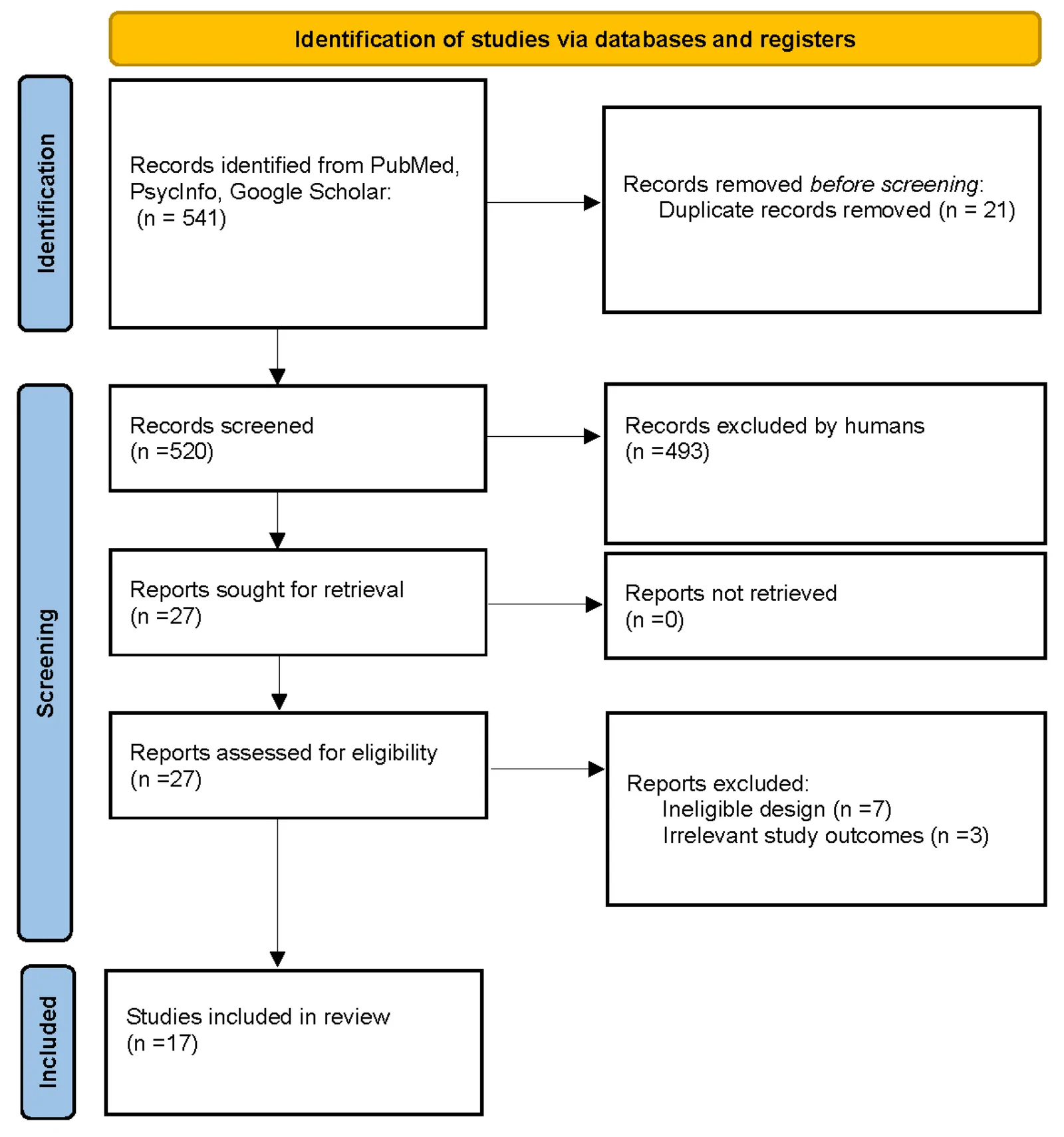
PRISMA Diagram of Identification Process for Eligible Studies (*n* = 17).

Our search strategy generated a total of 541 potentially eligible articles; however, 21 duplicate articles were removed. After title and abstract screening, 493 articles were removed for not meeting the inclusion criteria, and 27 were sought for retrieval. Following full-text review, seven articles were excluded for incorrect study design and three articles were excluded for incorrect outcomes. A total of 17 articles were found eligible after a thorough review of the identified literature. Figure 1 was created using PRISMA 2020 flow diagram to illustrate the selection process.

All eligible studies were conducted in the United States between 2018 and 2025. Among all eligible studies, one focused on adolescents aged 15 to 18 years old, while the majority of them were conducted with adults over the age of 18 years old. The sample size ranged from 410 to 12,557 participants, mostly targeting smokers. A majority of the studies differentiated participants by smoking status, including current smokers, dual-users (individuals who reported using more than one combustible and/or non-combustible tobacco product simultaneously), non-smokers, and former smokers. All studies were conducted online, with a majority classified as randomized controlled trials with one or more conditions. Of the 17 included studies, [3] were rated Good quality, [8] Fair, and [2] Poor based on the NIH quality assessment. The most common sources of methodological concern were the use of online convenience samples with uncertain representativeness, absence of blinding for outcome assessment, and reliance on immediate post-exposure measurement without longitudinal follow-up. These quality characteristics are noted where relevant in the sections below and should be considered when interpreting the consistency of findings across studies. Table S4 in the Supplementary Materials presents a summary of all included studies.

All the studies were reviewed and assessed based on study outcomes. Analysis of the review revealed two categories, and the studies were grouped accordingly based on study characteristics and main outcomes. The first category included seven studies that concentrated on providing education about nicotine’s potential health risk (“nicotine education”) versus information about nicotine’s potential for health risk as it differs across delivery methods (nicotine continuum of risk (“COR”) education). Whereas the second category concentrated on ten studies that focused on measuring participants’ changes in knowledge, beliefs, and perceptions regarding either nicotine or the relative risk of nicotine products. The summarized study outcomes for Category 1 are presented in Table S5, while those for Category 2 are shown in Table S6 of the Supplementary Materials.

### 3.1. Category 1: Studies That Focused on Providing Nicotine Education and Its Impact on Knowledge, Perceptions and Behavioral Intentions

The seven studies in this category share a common intervention logic: exposing participants to brief educational content designed to correct widely-held misperceptions about nicotine—primarily the false belief that nicotine itself is the primary driver of smoking-related cancers and disease. Across the studies, message content consistently addressed nicotine’s addictive but non-carcinogenic properties, the continued risks associated with combustible tobacco, and the relative risk profiles of alternative products including reduced nicotine content (RNC) cigarettes, electronic nicotine delivery systems (ENDS), and nicotine replacement therapies (NRTs). While most studies used general corrective messaging, one study compared industry-proposed advertising with focused educational content [28], and one developed targeted messages for populations disproportionately affected by smoking, including Black/African American adults, rural adults, and young adults aged 18–25 [29].

Knowledge and perception outcomes were consistently positive across all seven studies, regardless of message format or framing. Three key patterns emerge from this body of work. First, corrective messaging targeting the nicotine-cancer misperception was particularly robust: Villanti et al. (2019) [30] found that brief nicotine education more than doubled the probability of a correct response to items about nicotine’s role in cancer, and subsequent replication with a larger, repeated-exposure design yielded similar results [31]. Second, product-specific misconceptions—particularly about reduced-risk products—were also amenable to correction, though with variability. Shi et al. (2024) [32] reported that VLNC-focused messages consistently reduced agreement with the misbelief that VLNC cigarettes are healthier than conventional cigarettes (M = 2.55, SD = 1.29 vs. M = 3.10, SD = 1.10 in controls) across all four tobacco use statuses; however, Mercincavage et al. (2024) [20] found that only one of several tested messages significantly improved accurate beliefs about RNC cigarette harms, suggesting that message specificity and framing matter. Third, the source and framing of messages modulates outcomes: Mercincavage et al. (2023) [28] found that both industry-proposed and focused educational messages reduced false beliefs about advertised products among non-smokers, though smokers were more resistant to belief change—a pattern consistent with motivated reasoning among those who have a vested interest in the product.

Behavioral intention outcomes were more mixed and warrant careful interpretation. Two studies did not assess behavioral outcomes at all [20,29], limiting what can be concluded about downstream effects from those interventions. Among the five studies that did measure intentions, a consistent pattern emerged: knowledge gains did not reliably translate into changed behavioral intentions to switch products or reduce use. Two studies found no significant shifts in intentions to use alternative nicotine products following exposure [28,31], and Yang et al. (2019) [33] similarly found no change in e-cigarette use intentions, though intentions to seek information about e-cigarettes did increase—a potentially important precursor to behavior change that deserves further investigation. The one study that did find a behavioral signal was Shi et al. (2024) [32], where dual/poly cigarette smokers reported significantly greater intentions to use VLNC cigarettes compared to exclusive cigarette smokers (*p* < 0.01), suggesting that tobacco use status may moderate the relationship between corrective education and behavioral intentions. Taken together, these findings indicate that nicotine education reliably improves knowledge and reduces misperceptions, but that the pathway from corrected beliefs to changed behavior is not automatic and is likely moderated by individual-level factors including use status, prior beliefs, and nicotine dependence.

### 3.2. Category 2: Studies Focused on COR Education and Its Impact on Knowledge, Perceptions and Behavioral Intentions

Ten studies examined the effects of educational messaging comparing the risks of combustible and noncombustible tobacco products. Nine studies enrolled adult participants (18+); one focused on adolescents aged 15–18 [34]. Participants included current and former smokers, non-smokers, dual-users, and e-cigarette users. Most studies employed online RCT designs (*n* = 9); one used a pre-post intervention design without a control group [27]. Products addressed included combustible cigarettes, ENDS (including JUUL), VLNC cigarettes, and RNC cigarettes. Four thematic patterns emerged across this category.

#### 3.2.1. Knowledge and Risk Perception: Consistent Gains Across Formats

Across all ten studies, exposure to comparative risk messaging was associated with improved knowledge and more accurate risk perceptions to some degree, regardless of format, population, or specific product addressed. Participants who received relative risk information were consistently more likely to accurately recognize that e-cigarettes carry lower but not zero risk compared to combustible cigarettes [18,35,36,37,38]. This pattern held across diverse delivery modalities, suggesting that the informational content itself, rather than any particular format, is the primary driver of knowledge gains. The single adolescent-focused study confirmed that this effect extends to younger audiences: Instagram-style messages highlighting e-cigarette harms and industry practices significantly increased both knowledge and accurate beliefs about e-cigarettes (*p* < 0.001) [34], with nearly 80% of adolescent participants reporting willingness to share the messages with peers, a potential amplification mechanism worth exploring in future research.

#### 3.2.2. The Role of Emotional Framing: Hope, Fear, and Anger as Behavioral Levers

Three studies [39,40,41] investigated how emotional responses to comparative risk messages shape downstream outcomes, and their findings offer some of the most actionable guidance for message designers. Yang et al. (2019) [39] found that hope and fear elicited by comparative risk messages were positively associated with intentions to quit smoking and seek cessation support, while disgust was associated with greater intentions to switch to e-cigarettes. Anger, however, produced the opposite effect: it was associated with reduced perceived cigarette risk and lower quit intentions, suggesting that adversarial or accusatory framing may backfire. Yang et al. (2019) [40] extended this by comparing positive comparative risk (CR) and negative comparative risk (CR−) message conditions: CR messages reduced perceived e-cigarette risk, CR− messages increased self-efficacy to quit, but both conditions reduced intention to smoke cigarettes and increased intent to switch to e-cigarettes. This convergence across valence conditions is notable, suggesting that the comparative risk content, not just the emotional appeal, is driving behavioral intentions. Yang et al. (2019) [41] further found that smokers with serious psychological distress (SPD) were more motivated to use e-cigarettes to aid cessation, pointing to the potential relevance of mental health status as a moderator of message effects that warrants dedicated investigation.

#### 3.2.3. Format and Delivery Channel: Broader Reach, Modest Differential Effects

Message format varied considerably across the ten studies, encompassing video advertisements [36] (This study was funded by JUUL Labs, Inc., Washington, D.C., USA), infographics [27], mock social media interfaces [34], text-based paragraphs [35], fact sheets [38], and colored imagery [39,40,41]. While all formats produced knowledge gains, evidence for differential effectiveness by format is limited. The infographic used by Leavens et al. (2021) [27] produced the largest knowledge gains among participants who had never used JUUL or smoked, suggesting that format accessibility may matter most for audiences with low baseline familiarity. The mock Instagram interface used by Lazard (2021) [34] is notable as the only format designed specifically for adolescents, and its success in significantly increasing accurate beliefs (*p* < 0.001) supports further investment in platform-native digital delivery for youth audiences. The industry-funded video study (McCaffrey et al., 2025 [36] (This study was funded by JUUL Labs, Inc.)) found that nearly 90% of smokers correctly understood that switching to JUUL would reduce but not eliminate toxic exposure, and that JUUL carries its own risks. While these comprehension rates are encouraging, this study’s funding by JUUL Labs, Inc. introduces the potential for outcome reporting favorable to the sponsor’s product [42], and these findings should be interpreted with appropriate caution.

#### 3.2.4. Behavioral Intentions: Conditional and Moderated Effects

Behavioral intention outcomes were more variable than knowledge outcomes, and the pattern of findings suggests that COR education is most likely to influence intentions under specific conditions. Mumford et al. (2019) [18] found that adult smokers were more likely than non-smokers to report intentions to use less harmful e-cigarette products following exposure (*p* < 0.001), pointing to smoking status as a key moderator. Yang et al. (2020) [38] found that comparative risk messages, particularly when combined with a nicotine fact sheet, improved self-efficacy beliefs about switching to e-cigarettes, suggesting that pairing product comparison with accurate nicotine information addresses both the “why” and the “how” of product substitution. Pei et al. (2025) [37] demonstrated that dual users in the VLNC message condition had higher intentions to switch to e-cigarettes in the next 6 months compared to other message conditions, again indicating that use status moderates’ responsiveness. Conversely, Yang et al. (2018) [35] found that targeted messages about ENDS did not produce better health-related beliefs or quit intentions than non-targeted messages, a null finding that highlights the limits of targeting by smoker type alone without incorporating emotional or motivational elements. Taken together, these findings suggest that COR messaging is most likely to shift behavioral intentions when it is paired with emotional engagement, combined with nicotine-specific information, and tailored to current tobacco use status.

## 4. Discussion

Ongoing literature emphasizes the lack of knowledge and/or false beliefs possessed by smokers and non-smokers, pertaining to nicotine itself and nicotine and/or tobacco products, which contributes to smoking prevalence remaining a critical public health issue in the U.S. Despite increased public health efforts, gaps remain in understanding how nicotine and relative risk education impact awareness, perceptions, and behavioral intentions among adolescents and adult smokers. This scoping review aimed to address this gap by synthesizing existing research on the efficacy of providing educational messaging and examining its influence on knowledge, beliefs, and changes in smoking behaviors, including switching to alternative tobacco products that pose lower risks and health harms such as ENDS and NRT. Overall, the studies suggest educational interventions can benefit smokers and nonsmokers by providing factual information regarding the risks and harms of nicotine and various nicotine and/or tobacco products, correcting any prior misconceptions and evoking a potential behavioral response.

Across both categories of studies, findings were consistently stronger for knowledge and risk perception outcomes than for behavioral intentions or use behaviors, a pattern consistent with the well-documented knowledge–attitude–behavior gap in health communication research [43]. The ability of a single, short-term educational exposure to shift health behavior is inherently limited: behavior change typically requires repeated exposure, social reinforcement, and structural support that extend well beyond what a controlled messaging experiment can provide. This gap between what these studies can illustrate and what policymakers need to know about real-world impact is an important limitation of the current evidence base, and future work should prioritize longitudinal designs and behavioral economic measures such as purchasing tasks or simulated product choice to better capture whether knowledge gains translate into meaningful decisions.

It is also important to recognize that factors beyond message exposure itself likely shape the degree to which educational interventions improve knowledge and influence behavior. Across the included studies, tobacco use status consistently emerged as a moderating factor, with current smokers, dual-users, and non-users often responding differently to the same messaging content [18,32]. Health literacy, sociodemographic characteristics such as race/ethnicity and socioeconomic status, and prior beliefs about nicotine also likely influence receptivity to educational messages and the translation of knowledge gains into behavioral intentions, though few studies were designed to formally test these moderating pathways [9]. Emotional responses to messaging—particularly hope, fear, and disgust—were also identified as mechanisms through which educational content may influence intentions, suggesting that message design features interact with individual-level factors to shape outcomes [39].

Integration of nicotine education within broader relative risk frameworks appears to be helpful in increasing awareness across a wide range of delivery modalities, including text-based messages [20,35], fact sheets [32,33,38], infographics [27], images [29,30,31,37,39,40,41], advertisements [28], videos [36] (This study was funded by JUUL Labs, Inc.), and depiction of mock social media interface [34]. Importantly, all studies required participants to attend to the educational content, indicating that capturing attention is a key mechanism of impact. Additionally, two studies incorporated factors of emotional and curiosity-eliciting elements within their educational messages to determine how that influences participants’ cognitive engagement and attention to educational material, motivation to change smoking behaviors, and shifts in knowledge [29,39]. These findings highlight the potential of employing these strategies to enhance the overall impact of educational communications. 

Investigators across the studies tested the educational exposure in various populations including smokers, non-tobacco or nicotine users, prior smokers, and dual-users, suggesting broad applicability of educational messaging approaches. However, nearly all studies only assessed knowledge and behavioral intentions immediately upon educational exposure. Future research should evaluate pre-exposure knowledge and behavioral intentions, measure immediate post-exposure changes in knowledge and behavioral intentions, and include long-term follow-up to determine whether correcting misconceptions and increasing awareness led to sustained reductions in smoking or adopting harm reduction strategies over time. The findings of the studies that concentrated on assessing changes in tobacco-related behaviors reported mixed results regarding intentions following educational exposure.

An important but understudied risk of nicotine and relative risk educational messaging is the potential for boomerang effects, in which corrective information inadvertently reinforces unintended beliefs or behaviors [44]. For example, messaging that emphasizes the comparative safety of ENDS relative to cigarettes may, for some audiences, reduce overall concern about nicotine use rather than motivating cessation or product switching. None of the 17 studies included in this review were designed to detect boomerang effects, and future research should prospectively evaluate whether and for whom relative risk messaging produces unintended consequences.

The study participants are not fully representative of the U.S. population, limiting generalizability. Underrepresented groups, such as Hispanic/Latino communities and sexual and gender minorities, experience disproportionate tobacco-related harms [45,46] and were not exclusively targeted as participants for the included studies of this scoping review. Only one eligible study employed a targeted messaging approach towards Black/African American, rural adults and young adults [29]. The results revealed that rural adults responded differently from Black/African American adults and young adults to curiosity-eliciting message components. These findings highlight the importance of targeted and culturally responsive educational approaches that are needed to address these gaps and ensure equitable public health impact. Additionally, only one study [41] recruited current and former smokers, with and without SPD, to examine their responses to comparative risk messages regarding e-cigarettes and cigarettes.

The utility of risk perception as an intervention target is also likely to vary by age and vulnerability. Among adolescents, risk perception may be less predictive of behavior given developmental factors such as present-bias and susceptibility to social influence [47]. Among adults with lower health literacy or higher nicotine dependence, the relationship between corrected risk perception and behavior change may also be attenuated. The single adolescent-focused study in this review [34] found that messaging improved knowledge and beliefs but did not assess downstream behavioral outcomes, underscoring the need for age-stratified research designs that can capture how risk perception functions differently across populations.

### 4.1. Limitations

#### 4.1.1. Limitations of Eligible Studies

Although formal risk-of-bias assessment is not required for scoping reviews, it is worth noting that the included studies varied considerably in methodological rigor. Several relied on convenience samples recruited via online platforms, which may limit representativeness, and the majority assessed outcomes immediately post-exposure without follow-up, making it difficult to draw conclusions about the durability of effects. These design features should be considered when interpreting the consistency of knowledge and perception outcomes reported across studies. These findings support the necessity of future studies to incorporate longitudinal designs that can help identify strategies most likely to impact behavior over time, assess changes in hypothetical or observed demand based on exposure to education, and assess any potential “boomerang effect,” are all critical to inform effective policy development. Notably, the eligible studies were predominantly based on adult samples, with limited evidence among adolescents; only one study [33] focused specifically on this age group. This highlights a critical gap that should be addressed by future research to improve adolescents’ understanding of the risks associated with various tobacco products.

#### 4.1.2. Limitations of the Present Study

This study is limited by the scope of the databases and search strategy. The initial search only relied on Google Scholar and PubMed, using a combination of relevant keywords without MeSH terms. This approach may have resulted in the omission of relevant studies. However, the search strategy was later revised to include PsycINFO, utilizing both MeSH terms and additional keywords to improve the coverage of the search. Future studies should adopt a comprehensive search strategy from the outset and include multiple databases, such as Scopus and Web of Science, to ensure broader coverage of the literature. Additionally, during the initial search strategy, abstract and title screening was conducted by a single author (A.P.), which may have introduced selection bias. This process was later revised so that two authors (A.P. and R.I.) independently completed the initial screening. Any discrepancies were subsequently resolved through discussion among all authors to improve reliability.

One methodological limitation concerns the timing of protocol registration. Although the protocol for this review was registered on the Open Science Framework (OSF) on 2 April 2026, the updated literature search was conducted in March 2026, as both actions resulted from responding to the first round of peer review. Because registration occurred after the search, and potentially after initial review of results, it did not serve its intended purpose of preventing hindsight bias in methodological decision-making. We acknowledge this as a limitation on the transparency of the review process and recommend that future reviews in this area preregister protocols prior to initiating searches.

## 5. Conclusions

This scoping review synthesizes evidence indicating that educational messaging about nicotine risks and harms is associated with improved knowledge and reduced misperceptions across a range of populations and delivery formats; however, the predominantly fair methodological quality of included studies and their reliance on immediate post-exposure outcomes limit the strength of conclusions that can be drawn about durable behavior change. When creating educational messages and interventions, it can be beneficial to employ strategies that capture their attention and are easily comprehended to maximize the impact of these materials. It is imperative to develop targeted interventions, especially for populations that are disproportionately affected by smoking. Educational materials that are tailored and relevant to individuals’ unique lived experiences may increase the likelihood of shifting smoking behaviors and reduce harm. Future work needs to be centered around a longitudinal study design to exhibit how educational strategies translate into meaningful behavior change. Ultimately, expanding access to accurate and accessible educational resources across the U.S. population has the potential to reduce the national burden of smoking.

## Data Availability

The original contributions presented in this study are included in the article. Further inquiries can be directed to the corresponding author.

## Supplementary Materials

The following supporting information can be downloaded at https://www.mdpi.com/article/10.3390/ijerph23050636/s1. Table S1. Preferred Reporting Items for Systematic reviews and Meta-Analyses extension for Scoping Reviews (PRISMA-ScR) Checklist; Table S2. Search Strategy for Included Databases. Note: Work on this manuscript began in September of 2024 where initial searches were performed using Google Scholar and PubMed to identify potentially eligible articles. In March 2026, we updated our search with a more robust strategy that utilized MeSH terms to capture all potentially eligibly articles; Table S3. National Institutes of Health (NIH) Risk of Bias (ROB) assessment for eligible studies. Note: A formal quality appraisal for 16 of the included studies was conducted using the NIH Study Quality Assessment Tool for Controlled Intervention Studies [26], with the Quality Assessment Tool for Before-After (Pre-Post) [26] applied to Leavens et al. (2021) [27]. Legends: Y = Yes; N = No; N/A = Not applicable; ? = Unclear or not reported; G = Good; F = Fair; P = Poor; † Use Before–After tool; ‡ Industry-funded studies were considered as having additional risk; Table S4. Selected characteristics of studies eligible for inclusion (*n* = 17); Table S5. Studies focused on the associations between nicotine educational message exposure, and knowledge, perceptions, and behavioral intentions (*n* = 7); Table S6. Studies examining associations between relative risk educational messaging and knowledge, perceptions, and behavioral intentions (*n* = 10).

## Abbreviations

The following abbreviations are used in this manuscript:

NRT	Nicotine Replacement Therapy
ENDS	Electronic Nicotine Delivery Systems
COR	Continuum of Risk
RNC	Reduced Nicotine Content
VLNC	Very Low Nicotine Content
RCT	Randomized Controlled Trial
SPD	Serious Psychological Distress
