# Correction: Imran et al. Nicotine Risk Education and Its Impact on Knowledge, Perceptions, and Behavioral Intentions: A Scoping Review of U.S. Studies. *Int. J. Environ. Res. Public Health* 2026, *23*, 636

**DOI:** 10.3390/ijerph23070939

**Published:** 2026-07-22

**Authors:** Rabia Imran, Aashka Patel, Morgan Snell

**Affiliations:** 1 Department of Health Policy, Virginia Commonwealth University, Richmond, VA 23284, USA; imranr@vcu.edu (R.I.); patelas13@vcu.edu (A.P.); 2 Department of Biology, Virginia Commonwealth University, Richmond, VA 23284, USA

In the original publication [1], there was an error in the citation supporting the following sentence:

“Importantly, holding these inaccurate beliefs about nicotine may reduce the likelihood that smokers will attempt to quit or adopt less harmful alternatives such as NRTs or ENDS to aid cessation or replace cigarettes [8].”

The original Reference [12] has been replaced with a more relevant Reference [8]. The original Reference [12] has been removed, and the subsequent references have been renumbered accordingly.

The authors state that the scientific conclusions are unaffected. This correction was approved by the Academic Editor. The original publication has also been updated.

## References

[1] Imran R., Patel A., Snell M. (2026). Nicotine Risk Education and Its Impact on Knowledge, Perceptions, and Behavioral Intentions: A Scoping Review of U.S. Studies. Int. J. Environ. Res. Public Health.

