# Supplementary material for: Nicotine Risk Education and Its Impact on Knowledge, Perceptions, and Behavioral Intentions: A Scoping Review of U.S. Studies
Source: Int J Environ Res Public Health. 2026 May 11;23(5):636. doi: 10.3390/ijerph23050636 (PMC13207396; doi:10.3390/ijerph23050636)
Supplement: Supplementary file 1 [file ijerph-23-00636-s001.zip › ijerph-4175854-supplementary.pdf]

Supplementary Materials

Table S1. Preferred Reporting Items for Systematic reviews and Meta-Analyses extension for Scoping Reviews (PRISMA-ScR) Checklist.

SECTION	ITEM	PRISMA-ScR CHECKLIST ITEM	REPORTED ON PAGE #
**TITLE**			
Title	1	Identify the report as a scoping review.	1
**ABSTRACT**			
Structured summary	2	Provide a structured summary that includes (as applicable): background, objectives, eligibility criteria, sources of evidence, charting methods, results, and conclusions that relate to the review questions and objectives.	1-2
**INTRODUCTION**			
Rationale	3	Describe the rationale for the review in the context of what is already known. Explain why the review questions/objectives lend themselves to a scoping review approach.	2-4
Objectives	4	Provide an explicit statement of the questions and objectives being addressed with reference to their key elements (e.g., population or participants, concepts, and context) or other relevant key elements used to conceptualize the review questions and/or objectives.	2-4
**METHODS**			

Protocol and registration	5	Indicate whether a review protocol exists; state if and where it can be accessed (e.g., a Web address); and if available, provide registration information, including the registration number.	4
Eligibility criteria	6	Specify characteristics of the sources of evidence used as eligibility criteria (e.g., years considered, language, and publication status), and provide a rationale.	4
Information sources*	7	Describe all information sources in the search (e.g., databases with dates of coverage and contact with authors to identify additional sources), as well as the date the most recent search was executed.	4
Search	8	Present the full electronic search strategy for at least 1 database, including any limits used, such that it could be repeated.	4
Selection of sources of evidence†	9	State the process for selecting sources of evidence (i.e., screening and eligibility) included in the scoping review.	5
Data charting process‡	10	Describe the methods of charting data from the included sources of evidence (e.g., calibrated forms or forms that have been tested by the team before their use, and whether data charting was done independently or in duplicate) and any processes for obtaining and confirming data from investigators.	5
Data items	11	List and define all variables for which data were sought and any assumptions and simplifications made.	5
Critical appraisal of individual sources of evidence§	12	If done, provide a rationale for conducting a critical appraisal of included sources of evidence; describe the methods used and how this information was used in any data synthesis (if appropriate).	5
Synthesis of results	13	Describe the methods of handling and summarizing the data that were charted.	5-7

**RESULTS**			
Selection of sources of evidence	14	Give numbers of sources of evidence screened, assessed for eligibility, and included in the review, with reasons for exclusions at each stage, ideally using a flow diagram.	5-7
Characteristics of sources of evidence	15	For each source of evidence, present characteristics for which data were charted and provide the citations.	5-6 and Table S4 (Supplementary Materials)
Critical appraisal within sources of evidence	16	If done, present data on critical appraisal of included sources of evidence (see item 12).	6
Results of individual sources of evidence	17	For each included source of evidence, present the relevant data that were charted that relate to the review questions and objectives.	7-9
Synthesis of results	18	Summarize and/or present the charting results as they relate to the review questions and objectives.	7-9
**DISCUSSION**			
Summary of evidence	19	Summarize the main results (including an overview of concepts, themes, and types of evidence available), link to the review questions and objectives, and consider the relevance to key groups.	9-11
Limitations	20	Discuss the limitations of the scoping review process.	11-12
Conclusions	21	Provide a general interpretation of the results with respect to the review questions and objectives, as well as potential implications and/or next steps.	12
**FUNDING**			

Funding	22	Describe sources of funding for the included sources of evidence, as well as sources of funding for the scoping review. Describe the role of the funders of the scoping review.	13

Table S2. Search Strategy for Included Databases.

#	Search Terms	Database	Date of Search
1	“Nicotine OR tobacco” AND “education” AND “perceptions OR attitudes OR knowledge”	PubMed	September 29, 2024
2	nicotine AND education AND harm AND message	PubMed	September 29, 2024
3	("nicotine" OR "tobacco") AND ("education" OR "message") AND harm	PubMed	September 29, 2024
4	“Nicotine OR tobacco” AND “educational messages” AND “misperceptions”	Google Scholar	October 5, 2024
5	("nicotine" OR "tobacco") AND "educational message" AND ("harm" OR "risk perceptions") AND message	Google scholar	October 5, 2024
6	"nicotine reduction" AND "educational messages" AND harm	Google Scholar	October 5, 2024
7	"Comparative" AND ("risk" OR "harm") AND ("nicotine" OR "tobacco") AND ("message" OR "education")	PubMed	October 14, 2024
8	("Electronic Nicotine Delivery Systems"[MeSH] OR "e-cigarette*"[tiab] OR ENDS[tiab] OR vape*[tiab]) AND ("Harm Reduction"[MeSH] OR "relative risk"[tiab] OR "comparative risk"[tiab] OR "continuum of risk"[tiab]) AND ("Intention"[MeSH] OR "behavioral intention*"[tiab] OR "quit intention*"[tiab] OR "smoking cessation"[MeSH])	PubMed	March 30, 2026
9	(TI "electronic nicotine delivery system*" OR AB "electronic nicotine delivery system*" OR TI "e-cigarette*" OR AB "e-cigarette*" OR TI ENDS OR AB ENDS OR TI vape OR AB vape) AND (SU "Harm Reduction" OR TI "harm reduction" OR AB "harm reduction" OR TI "relative risk" OR AB "relative risk" OR TI "comparative risk" OR AB "comparative risk" OR TI "continuum of risk" OR AB "continuum of risk") AND (SU "Intentions" OR TI "behavioral intention*" OR AB "behavioral	PsycInfo	March 30, 2026

	intention*" OR TI "quit intention*" OR AB "quit intention*" OR TI intention OR AB intention) AND (SU "Smoking Cessation" OR TI "smoking cessation" OR AB "smoking cessation")		
10	("Electronic Nicotine Delivery Systems"[MeSH] OR "e-cigarette*"[tiab] OR ENDS[tiab] OR vape[tiab]) AND ("Harm Reduction"[MeSH] OR "relative risk"[tiab] OR "comparative risk"[tiab] OR "continuum of risk"[tiab]) AND ("Intention"[MeSH] OR "behavioral intention*"[tiab] OR "quit intention*"[tiab] OR "smoking cessation"[MeSH]) AND ("Health Education"[MeSH] OR education[tiab] OR "health message*"[tiab] OR message*[tiab] OR "public health campaign*"[tiab])	PubMed	March 31, 2026
11	(SU "Electronic Nicotine Delivery Systems" OR TI(e-cigarette* OR ecigarette* OR "electronic cigarette*" OR vape* OR ENDS) OR AB(e-cigarette* OR ecigarette* OR "electronic cigarette*" OR vape* OR ENDS)) AND (SU "Harm Reduction" OR TI("relative risk" OR "comparative risk" OR "continuum of risk") OR AB("relative risk" OR "comparative risk" OR "continuum of risk")) AND (SU "Smoking Cessation" OR SU "Intentions" OR TI("behavioral intention*" OR "quit intention*" OR "smoking cessation") OR AB("behavioral intention*" OR "quit intention*" OR "smoking cessation")) AND (SU "Health Education" OR TI(education OR message* OR "health message*" OR "public health campaign*") OR AB(education OR message* OR "health message*" OR "public health campaign*"))	PsycInfo	March 31, 2026

Note: Work on this manuscript began in September of 2024 where initial searches were performed using Google Scholar and PubMed to identify potentially eligible articles. In March 2026, we updated our search with a more robust strategy that utilized MeSH terms to capture all potentially eligibly articles.

Table S3. National Institutes of Health (NIH) Risk of Bias (ROB) assessment for eligible studies.

Study	Q1	Q2	Q3	Q4	Q5	Q6	Q7	Q8	Q9	Q10	Q11	Q12	Q13	Q14	Overall (G/F/P)
**Category 1: Nicotine Education Studies**															
Mercincavage et al. (2023) [29]	Y	N	N/A	N/A	N/A	Y	Y	?	Y	Y	Y	Y	Y	N	**Fair**
Mercincavage et al. (2024) [21]	Y	N	N/A	N/A	N/A	Y	N/A	N/A	Y	Y	Y	?	Y	Y	**Fair**
Shi et al. (2024) [33]	Y	N	N/A	N/A	N/A	Y	N/A	N/A	Y	Y	Y	N	Y	?	**Poor**

Study	Q1	Q2	Q3	Q4	Q5	Q6	Q7	Q8	Q9	Q10	Q11	Q12	Q13	Q14	Overall (G/F/P)
Villanti et al. (2019) [31]	Y	N	N/A	N/A	N/A	Y	N/A	N/A	Y	Y	Y	?	Y	Y	**Fair**
Villanti et al. (2025) [32]	Y	Y	N/A	N/A	N/A	Y	N	Y	Y	Y	Y	Y	Y	Y	**Good**
Wang et al. (2025) [30]	Y	N	N/A	N/A	N/A	Y	N/A	N/A	Y	Y	Y	Y	Y	Y	**Fair**
Yang et al. (2019) [34]	Y	N	N/A	N/A	N/A	Y	N/A	N/A	Y	Y	Y	?	Y	Y	**Fair**
**Category 2: COR Education Studies**															
Lazard (2021) [35]	Y	N	N/A	N/A	N/A	Y	N/A	N/A	Y	Y	Y	?	?	Y	**Fair**
Leavens et al. (2021) † [28]	Y	Y	?	?	Y	Y	Y	N/A	?	Y	N	N/A	-	-	**Good**
Mumford et al. (2019) [19]	Y	N	N/A	N/A	N/A	Y	N/A	N/A	Y	Y	Y	?	Y	Y	**Fair**
McCaffrey et al. (2025) ‡ [37]	Y	N	N/A	N/A	N/A	Y	N/A	N/A	Y	Y	Y	?	?	Y	**Poor**
Pei et al. (2025) [38]	Y	Y	N/A	N/A	N/A	Y	Y	?	Y	Y	?	Y	Y	Y	**Good**
Yang et al. (2018) [36]	Y	N	N/A	N/A	N/A	Y	N/A	N/A	Y	Y	Y	?	Y	?	**Fair**
Yang et al. (2019) [40]	Y	N	N/A	N/A	N/A	Y	N/A	N/A	Y	Y	Y	?	Y	N	**Fair**
Yang et al. (2019) [42]	Y	N	N/A	N/A	N/A	Y	N/A	N/A	Y	Y	Y	?	Y	Y	**Fair**
Yang et al. (2019) [41]	Y	N	N/A	N/A	N/A	Y	N/A	N/A	Y	Y	Y	?	Y	Y	**Fair**
Yang et al. (2020) [39]	Y	N	N/A	N/A	N/A	Y	N/A	N/A	Y	Y	Y	?	Y	N	**Fair**

Y = Yes | N = No | N/A = Not Applicable | ? = Unclear/not reported | G = Good, F = Fair, P = Poor | † Use Before-After tool | ‡ Industry-funded: note as additional risk

Note: A formal quality appraisal for 16 of the included studies was conducted using the NIH Study Quality Assessment Tool for Controlled Intervention Studies [27], with the Quality Assessment Tool for Before-After (Pre-Post) [27] applied to Leavens et al. (2021) [28].

Table S4. Selected characteristics of studies eligible for inclusion (*n*=17).

Author/Year	Title	Population	Study Design	Education Focus	Study Outcomes	Major Findings
Mercincavage et al. (2023) [29]	Effects of advertising features on smokers' and non-smokers' perceptions of a reduced	n=807; adult (18+ in age) smokers and non-smokers	Online Randomized Controlled Trial (RCT)	Nicotine	Perceptions of health risks and beliefs	Participants in the treatment arm perceived greater addiction risk and greater health risks p<0.05.

Author/Year	Title	Population	Study Design	Education Focus	Study Outcomes	Major Findings
	nicotine cigarette modified risk tobacco product					
Mercincavage et al. (2024) [21]	Associations of educational and marketing messages with beliefs about nicotine and reduced nicotine cigarettes	n=2962; US adult (18-45 years old) smokers	Online RCT	Nicotine	Belief outcomes	Message sources were linked with higher likelihood of accurate beliefs about nicotine (p's < 0.01).
Shi et al. (2024) [33]	Correcting misperceptions about very low nicotine cigarettes for cigarette-only smokers, dual/poly smokers, other tobacco users, and non-tobacco users	n=410; US adult (18+ in age) non-tobacco users, cigarette only smokers, dual/poly smokers, other tobacco users	Online RCT	Nicotine	Tobacco use status; nicotine risk perception; behavioral beliefs of VLNC; VLNC attitude; VLNC intention	Participants in the treatment arm had reduced nicotine false beliefs and were less prone to holding the misconception of very low nicotine cigarettes being healthier than traditional combustible cigarettes.
Villanti et al. (2019) [31]	Impact of Brief Nicotine Messaging on Nicotine-Related Beliefs in a U.S. Sample	n=521; US adults (18+)	Online RCT	Nicotine	Nicotine, NRT, E-cigarette, and RNC cigarette beliefs; nicotine use; behavioral control regarding cigarette/tobacco use; intention to use cigarettes, NRT, E-cigarettes, and RNC cigarettes	Participants in the treatment arm had fewer false beliefs regarding nicotine and nicotine replacement therapy products ($p<0.001$), as well as, e-cigarettes and reduced nicotine content cigarettes ($p<0.05$).
Villanti et al. (2025) [32]	Effect of nicotine corrective messaging on nicotine-related beliefs in US adults: a randomized controlled trial	n=794; US adults (18+ in age)	Online RCT	Nicotine	Nicotine beliefs; use intentions; use of nicotine; tobacco products	Exposing participants to repeated nicotine corrective messages lead to reduced false beliefs regarding nicotine, nicotine replacement therapy, e-cigarettes and reduced nicotine content cigarettes.

Author/Year	Title	Population	Study Design	Education Focus	Study Outcomes	Major Findings
Wang et al. (2025) [30]	Targeting three United States priority populations of people who smoke with educational nicotine messages using curiosity-eliciting strategies	Adults (18+ in age) living in the US, identifying as one of the three populations (black/African American adults, rural adults and young adults) and are current smokers. Study 1 and 2 (*n* = 200 for each population)	Online Stratified Randomized Controlled Trial (SRCT)	Nicotine	Nicotine false beliefs (primary outcome), other measures included Relative Harms of Nicotine, Reduced Nicotine Cigarettes False Beliefs, E-Cigarettes False Beliefs, Nicotine Replacement Therapy False Beliefs and Perceived Message Effectiveness	Targeted messages seemed to be effective, among all three priority populations, in lowering nicotine false beliefs.
Yang et al. (2019) [34]	Effects of a Nicotine Fact Sheet on Perceived Risk of Nicotine and E-Cigarettes and Intentions to Seek Information About and Use E-Cigarettes	n=765; US adult current and recent former smokers	Online RCT	Nicotine	Perceived nicotine addictiveness; nicotine risk; comparative risk of e-cigarettes; dual use intentions	After viewing the nicotine fact sheet, participants were twice as likely to disagree that nicotine is the main reason behind smoking-related illnesses. Additionally, no significant change was observed concerning smoker's risk perception about e-cigarettes or intentions to switch.
Lazard (2021) [35]	Social Media Message Designs to Educate Adolescents About E-Cigarettes	n=928; US adolescent (15-18 years old) previous or current users	Online RCT	Relative Risk	E-cigarette knowledge and beliefs	Participants in the treatment arm presented greater knowledge ($p<.001$) and beliefs ($p<.001$) relating to the harms of e-cigarettes.
Leavens et al. (2021) [28]	Exploratory evaluation of online brief education for JUUL pod-mod use and prevention	n=947; US adults (18-30 years old) former and current smokers, current JUUL users, individuals that never smoked, individuals that never used JUUL	Online Intervention	Relative Risk	JUUL knowledge; harm perceptions; behavioral intentions; interest in use and susceptibility; motivation for change; brief education view time	The brief JUUL educational handout increased participants knowledge and risk perceptions regarding JUUL ($p \leq 0.001$). Additionally, results showed a decrease in participants interest in purchasing and regularly or occasionally using JUUL in the future ($p \leq 0.001$).

Author/Year	Title	Population	Study Design	Education Focus	Study Outcomes	Major Findings
Mumford et al. (2019) [19]	E-cigarette Beliefs: Testing a Relative Risk Message in a Representative US Sample	n=773; US adult (18+ in age) smokers and non-smokers	Online RCT	Relative Risk	E-cigarette risk perception; likelihood of e-cigarette use; tobacco product use	Participants had lower estimates of harm associated with e-cigarettes, and rated e-cigarettes as less harmful.
McCaffrey et al. (2025)[1] [37]	Randomized experimental test of a reduced-exposure message for an e-cigarette: comprehension and related misperceptions	n=12,557; US adults (18+ in age), current smokers, dual users of tobacco products, former smokers and non-smokers	Online randomized between-subjects experiment	Relative Risk	Message comprehension, absolute risk misperceptions, perception of intended audience, risk-taking and health literacy	Nearly 90% of the smokers that viewed the reduced-exposure message comprehended that switching to JUUL leads to less exposure. Additionally, the majority of the smokers and non-smokers did understand that JUUL comes with its own risks and does not completely eradicate exposure to toxic chemicals.
Pei et al. (2025) [38]	Independent and combined effects of very low nicotine cigarette messages and e-cigarette messages: a randomised clinical trial	n=1901; US adults (18+), exclusive cigarette smokers, dual users of e-cigarettes and combustible cigarettes and non-smokers	Online RCT	Relative Risk	Perceived absolute harm of very low nicotine cigarettes (VLNCs), perception of comparative harm of VLNC's (e.g., understanding of absolute and comparative addictiveness of VLNC's), perceived nicotine risks and intentions to quit cigarette smoking	Participants assigned to the VLNC condition had higher perceived harms of VLNCs compared to participants in the e-cigarette and control conditions.
Yang et al. (2018) [36]	Targeted Versus Nontargeted Communication About Electronic Nicotine Delivery Systems in Three Smoker Groups	n=580; US adult (18-64 years old) current smokers	Online RCT	Relative Risk	Perceived message effectiveness; message reactions; ENDS- and cigarette-related beliefs; behavioral intentions	Compared to non-targeted messages, the targeted messages were not effective in influencing absolute and comparative risk perception related to cigarettes and ENDS.

[1] This study was funded by JUUL Labs, Inc.

Author/Year	Title	Population	Study Design	Education Focus	Study Outcomes	Major Findings
Yang et al. (2019) [40]	Feeling Hopeful Motivates Change: Emotional Responses to Messages Communicating Comparative Risk of Electronic Cigarettes and Combusted Cigarettes	n=1202; US adults (18+ in age) current smokers or recent quitters	Online RCT	Relative Risk	Feelings of hope, happiness, fear, guilt, disgust, and anger; risk perception; behavioral intentions about e-cigarettes and cigarettes	Results found that integrating emotions, such as hope, fear, and guilt, in smoking-related messaging can lead to accurate absolute cigarette risks, raises intent to switch to e-cigarettes or use nicotine replacement therapy products and motivate individuals to seek help to quit smoking.
Yang et al. (2019) [42]	Psychological distress and responses to comparative risk messages about electronic and combusted cigarettes	n=1400; US adults (18+ in age) current smokers or recent former smokers, with or without serious psychological distress (SPD)	Randomized controlled online experiment	Relative Risk	Beliefs related to e-cigarette and cigarettes, and behavioral intentions	Findings demonstrate that cigarette smokers endorsing serious psychological distress (SPD) had higher intentions to fully switch to e-cigarettes and get support while quitting in comparison to smokers with no SPD.
Yang et al. (2019) [41]	Testing Messages About Comparative Risk of Electronic Cigarettes and Combusted Cigarettes	n=1400; US adults (18+ in age) current smokers or recent former smokers	Randomized controlled online experiment	Relative Risk	Intentions to switch to e-cigarettes, intentions for dual-use, future smoking intentions, perceived absolute risk of e-cigarettes, perception of relative harm, self-efficacy to quit cigarette smoking, behavioral intentions	Comparative risk messages increased smokers' intent to switch to less harmful tobacco products (e-cigarettes). Incorporating negative anti-smoking components in comparative risk communication may be beneficial, as they resulted in greater self-efficacy beliefs to quit cigarette smoking.
Yang et al. (2020) [39]	Communicating risk differences between electronic and combusted cigarettes: the role of the FDA-mandated addiction warning and a nicotine fact sheet	n=1528; US adult current smokers or recent quitters	Online RCT	Relative Risk	Message reactions; perceived effectiveness; e-cigarette-related and cigarette-related beliefs; behavioral intentions; nicotine-related beliefs	The nicotine fact sheet condition improved accurate beliefs regarding nicotine risk and self-efficacy related to switching to e-cigarettes.

Table S5. Studies focused on the associations between nicotine educational message exposure, and knowledge, perceptions, and behavioral intentions (*n*=7).

Author/Year	Population	Message Delivery/Attributes	Message(s) Content	Outcomes
Mercincavage et al. (2023) [29]	n=807; adult (18+ in age) smokers and non-smokers	Messages were displayed as a form of advertisement. White text was paired with a yellow background and relevant graphics, including 'surgeon general's warning' at the bottom.	Messages contained (1) Industry-proposed content; (2) Focused content; and (3) No content.	Non-smokers held less false beliefs regarding the advertised cigarettes than smokers. It was found that the disclaimer content significantly influenced non-smokers to have less false beliefs and more accurate health and risk perceptions concerning the advertised cigarettes compared to those that viewed ads with no content. Compared to non-smokers, smokers displayed an intent to buy and use advertised cigarettes if provided at no charge.
Mercincavage et al. (2024) [21]	n=2962; US adult (18-45 years old) smokers	Messages were presented as statements to participants. For example, "Some of the toxic chemicals in tobacco are present in the plant itself."	Message content included educational and marketing messages to correct beliefs about nicotine and nicotine delivery systems.	In comparison to reference messages, a total of five messages were linked with higher odds of holding accurate beliefs regarding the role of nicotine in causing cancer (all p's < 0.01). Additionally, only one message was associated with accurate beliefs about the harms of reduced nicotine cigarettes vs. the reference messages. No significant association was found among messages to correct beliefs regarding the addictiveness of reduced nicotine cigarettes compared to reference messages.
Shi et al. (2024) [33]	n=410; US adults (18+ in age) that are non-tobacco users, cigarette only smokers, dual/poly smokers, other tobacco users	Message was presented as a "Fact & Myths" sheet (Q&A format). The text was displayed on a white background and did not contain any visual graphics.	The message content consisted of information regarding nicotine and very low nicotine content cigarettes, it also included quitting resources specific to New York.	Across all tobacco use statuses, educational messages regarding very low nicotine cigarettes (VLNC) were effective in decreasing participants' risk perception about nicotine and lowered the misconception of VLNC being a healthier alternative than traditional cigarettes. The harms of VLNC second-hand smoke were reinforced among dual/poly cigarette smokers and other tobacco users. Attitude towards VLNC use was significantly greater among dual/poly and exclusive cigarette smokers. However, dual/poly cigarette smokers displayed greater intent to use VLNC compared to other groups.

Villanti et al. (2019) [31]	n=521; US adults (18+ in age)	The six messages presented to the participants in the nicotine education condition were displayed on a black slide with a visual of smoke, whereas participants in the control condition viewed six messages displayed on an orange slide with a visual of the sun.	Message content for the nicotine messaging condition included brief statements regarding harms and risks associated with using nicotine and how it could be used safely. Message content for the control condition were related to sun safety. The third condition showed no messages to the participants.	Exposure to nicotine educational messaging led to a decrease in false beliefs regarding nicotine, nicotine replacement therapy products, e-cigarettes, and reduced nicotine content cigarettes vs. the control conditions. It was also found that participants were twice as likely to accurately respond to the item 'nicotine is a cause of cancer.' There was no influence on belief regarding other substances present in a cigarette. Additionally, no impact was found on behavioral control and intentions to use various nicotine and/or tobacco products.
Villanti et al. (2025) [32]	n=794; US adults (18+ in age)	Six messages that were presented to participants in the study were adapted by Villanti et al. (2019), with two additional messages. A total of eight messages were viewed by participants, all displayed on a black background, written in white and yellow text and white smoke.	Message content was information regarding nicotine, nicotine replacement therapy products, e-cigarettes, and reduced nicotine content cigarettes.	Repetitive exposure to nicotine corrective messaging resulted in lower false beliefs regarding nicotine, e-cigarettes, nicotine replacement therapy products and reduced nicotine content cigarettes among participants. Findings suggest limited intervention effects on intentions and use of tobacco and/or nicotine products.
Wang et al. (2025) [30]	Adults (18+ in age) living in the US, identifying as one of the three populations (black/African American adults, rural adults and young adults) and are current smokers. Study 1 and 2 (*n* = 200 for each population).	The messages that were presented to participants in the study were adapted by Villanti et al. (2019). All messages were presented on a black slide and white smoke with text.	Message content consisted of brief statements regarding harms and risks associated with using nicotine and how it could be used safely.	Findings of this study demonstrate that different curiosity-eliciting features were effective in creating targeted effects among all three populations. Black/African American smokers and rural adult smokers had lower false beliefs about nicotine after viewing the targeted messages vs. standard messages. However, for young adult smokers, no significant differences were found between standard and targeted messaging.

Yang et al. (2019) [34]	n=765; US adult current and recent former smokers	Message was presented as a fact sheet with black text on a plain white background and no graphics. Some of the text was underlined and bold.	The nicotine fact sheet contained information regarding nicotine and its harmful and addictive properties.	Nicotine fact sheet accurately altered participants' misbelief of nicotine being the main reason behind smoking-related health diseases. No significant changes were found among smokers' risk perception regarding e-cigarettes and intentions to switch to them after viewing the nicotine fact sheet.

Table S6. Studies examining associations between relative risk educational messaging and knowledge, perceptions, and behavioral intentions (*n*=10).

Author/Year	Population	Message Delivery/Attributes	Message(s) Content	Outcomes
Lazard (2021) [35]	n=928; US adolescent (15-18 years old) previous or current users	Messages were paired with either positive images or negative images and were presented as a mock Instagram interface.	Message content included industry practices, as well as risks and harms associated with e-cigarettes.	Exposure to messages led to adolescents having more knowledge regarding e-cigarettes compared to those in the no-message condition ($p < .001$). Additionally, social media messages significantly impacted beliefs regarding the harms of e-cigarettes compared to the control condition. Nearly 80% of the participants reported wanting to share these educational messages with other youth.
Leavens et al. (2021) [28]	n=947; US adults (18-30 years old) former and current smokers, current JUUL users, individuals that never smoked, individuals that never used JUUL	Infographic with information about JUUL; colorful text and images on white background.	Message content consisted of a combination of various JUUL related facts.	Findings demonstrated an increase in participants' knowledge and harm perceptions related to JUUL after being exposed to the educational handout. The information was particularly impactful among participants that had never used JUUL nor smoked, as they exhibited the highest increase in knowledge. Participants displayed a decrease in their intent to use and purchase JUUL. Furthermore, educational handout increased participants commitment and willingness to quit JUUL.
Mumford et al. (2019)[19]	n=773; US adult (18+ in age) smokers and non-smokers	**N/A**	Message content for the test condition included a relative risk-message related to e-cigarettes whereas the control condition consisted of a general health	Participants in the test group perceived e-cigarettes with lower harm compared to those in the control group. Non-smokers in the test group, aged 30 and older, reported e-cigarettes to be less harmful vs. the non-smokers in the control group. Although an increase in participants'

			message which was not related to tobacco and/or nicotine product.	knowledge and beliefs was observed in the test group, no association was found in the probability of reported use of e-cigarettes in the future.
McCaffrey et al. (2025)[1] [37]	n=12,557; US adults (18+ in age), current smokers, dual users of tobacco products, former smokers and non-smokers	Messages were presented in a brief online video advertisement.	Message content included information about JUUL ENDS products. The test condition included a statement towards the end about reduced-exposure concerning JUUL ENDS product whereas the control condition did not include this statement.	The study found that after viewing the message, most participants held accurate perceptions about the risks associated with ENDS. Additionally, most recognized that switching to ENDS would diminish, but not eradicate, the harms of cigarette smoke. Nearly 90% of the intended audience (smokers) understood that quitting cigarettes completely would lead to reduced exposure.
Pei et al. (2025) [38]	n=1901; US adults (18+), exclusive cigarette smokers, dual users of e-cigarettes and combustible cigarettes and non-smokers	Messages for the VLNC condition were presented on a white or black background with text and graphic images. Messages for the e-cigarette condition were presented on a white background with colorful graphic images and text.	Messages for the VLNC condition contained information about the harms and risks associated with VLNCs, but each message indicated that they would be easier to quit. Message content in the e-cigarette condition focused on the reduced health risks for switching to e-cigarettes from combustible cigarette smoking. The control messages consisted of bottled water ads.	The results showed that participants in the VLNC and combined message conditions rated VLNCs as more harmful compared to those in the e-cigarette message condition. Intentions to switch to e-cigarettes were reported to be significantly higher among dual-using participants in the VLNC message condition as compared to the other three conditions. No significant differences were observed across message conditions, related to the perception of absolute addictiveness of VLNCs or the belief that they are less addictive than traditional cigarettes.
Yang et al. (2018) [36]	n=580; US adult (18-64 years old) current smokers	Messages were presented on a white screen with black text.	Message content was presented in a paragraph format with one message for each of the three smoker groups; content consisted of encouragement to quit smoking and information regarding incorporating ENDS as part of their quit plan.	Findings established that targeted messages did not result in better health-related beliefs, including overall and relative risk perceptions regarding ENDS and cigarettes, efficacy beliefs and quit intentions.
Yang et al. (2019) [40]	n=1202; US adults (18+ in age) current smokers or recent quitters	Messages were paired with background images related to the message content.	Messages were split into two categories: Comparative Risk Messages and Negative Comparative Risk messages.	Employing positive and negative emotions in communicating relative risks concerning modified risk tobacco products can be an effective approach. The study found that emotions such as hope, fear, and disgust led to

				greater intentions to quit smoking and seek aid for cessation. Happiness and guilt were associated with higher intent to use nicotine replacement therapy products and/or switch to e-cigarettes. While hope and disgust resulted in accurate risk perceptions, anger was associated with reduced perceived risks related to cigarettes and lower quit intentions.
Yang et al. (2019) [42]	n=1400; US adults (18+ in age) current smokers or recent former smokers, with or without serious psychological distress (SPD)	Comparative risk (CR) messages contained positive and colorful images and text, whereas the negative comparative risk (CR-) messages consisted of darker colors, negative imagery, and text. Participants were assigned to view one of the six risk messages (either type) or the control message.	The CR messages emphasized the health benefits of switching to e-cigarettes, whereas the CR- messages used negative anti-smoking elements, focusing on health harms and risks of cigarette smoking. Control messages consisted of bottled water advertisement.	Study findings established that, in comparison with smokers without SPD, smokers with SPD showed higher intentions of switching to e-cigarettes, sought assistance with quitting cigarettes, had more accurate perceptions of absolute risk for cigarettes and e-cigarettes, and were less likely to believe that e-cigarettes pose less harm than cigarettes.
Yang et al. (2019) [41]	n=1400; US adults (18+ in age) current smokers or recent former smokers	Same message attributes as above (Yang et al. 2019c). The only difference to be noted is that participants either viewed 1) all three CR messages, 2) all three CR- messages, or 3) the control message.	Same message content as above (Yang et al. 2019c).	Among smokers, both types of comparative risk messages led to reduced intentions to smoke cigarettes, motivated them to switch fully to e-cigarettes, and strengthened accurate perceptions of the relative harms of e-cigarettes. CR messages resulted in lowering the perception of absolute e-cigarette risks, while the CR- messages generated greater quitting self-efficacy.
Yang et al. (2020) [39]	n=1528; US adult current smokers or recent quitters	Messages were paired with relevant positive images and colorful text. The fact sheet consisted of black text against a plain white background.	Message content included Comparative Risk Messages, Comparative Risk Messages with Addiction Warnings, and Nicotine Fact Sheet.	Results revealed that participants across all message conditions reported accurate comparative risk perceptions of cigarettes and e-cigarettes and showed intent to switch to e-cigarettes. Participants who viewed the nicotine fact sheet were more likely to correctly understand risks associated with using nicotine and believed that switching to e-cigarettes could decrease health risks.
